# Hypoxia Decreases Invasin-Mediated *Yersinia enterocolitica* Internalization into Caco-2 Cells

**DOI:** 10.1371/journal.pone.0146103

**Published:** 2016-01-05

**Authors:** Nathalie E. Zeitouni, Petra Dersch, Hassan Y. Naim, Maren von Köckritz-Blickwede

**Affiliations:** 1 Department of Physiological Chemistry, University of Veterinary Medicine Hannover, Hannover, Germany; 2 Helmholtz Center for Infection Research, Braunschweig, Germany; 3 Research Center for Emerging Infections and Zoonoses (RIZ), University of Veterinary Medicine Hannover, Hannover, Germany; Duke University Medical Center, UNITED STATES

## Abstract

*Yersinia enterocolitica* is a major cause of human yersiniosis, with enterocolitis being a typical manifestation. These bacteria can cross the intestinal mucosa, and invade eukaryotic cells by binding to host β_1_ integrins, a process mediated by the bacterial effector protein invasin. This study examines the role of hypoxia on the internalization of *Y*. *enterocolitica* into intestinal epithelial cells, since the gastrointestinal tract has been shown to be physiologically deficient in oxygen levels (hypoxic), especially in cases of infection and inflammation. We show that hypoxic pre-incubation of Caco-2 cells resulted in significantly decreased bacterial internalization compared to cells grown under normoxia. This phenotype was absent after functionally blocking host β_1_ integrins as well as upon infection with an invasin-deficient *Y*. *enterocolitica* strain. Furthermore, downstream phosphorylation of the focal adhesion kinase was also reduced under hypoxia after infection. In good correlation to these data, cells grown under hypoxia showed decreased protein levels of β_1_ integrins at the apical cell surface whereas the total protein level of the hypoxia inducible factor (HIF-1) alpha was elevated. Furthermore, treatment of cells with the HIF-1 α stabilizer dimethyloxalylglycine (DMOG) also reduced invasion and decreased β_1_ integrin protein levels compared to control cells, indicating a potential role for HIF-1α in this process. These results suggest that hypoxia decreases invasin-integrin-mediated internalization of *Y*. *enterocolitica* into intestinal epithelial cells by reducing cell surface localization of host β_1_ integrins.

## Introduction

The human gastrointestinal (GI) tract is home to an array of bacteria, some commensals that are vital to human digestion and others that can cause acute or chronic infections. GI pathogens have been the subject of extensive studies, and many host-pathogen interactions in this tissue have been fully characterized. Thus, it is important to address the environmental setting in which these interactions occur and the factors that are involved. The GI tract represents its own microenvironment within the body: a vascularized, oxygenated, subepithelial mucosa bordered by the severely anoxic luminal region [1]. The intestinal epithelial layer has been shown to be in a physiological state of oxygen deprivation, also known as hypoxia, characterized by daily fluctuations in oxygen tensions with oxygen levels ranging from 1 to 7% [1–3]. This environment can be challenged even more upon onset of acute infections or chronic inflammation. In fact, infection sites often result in severe hypoxia, with oxygen levels dropping below 1% [4] because of decreased oxygen permeation, increased consumption by invading pathogens and infiltration of recruited immune cells [5,6]. Hypoxia has been shown to lead to numerous changes within host cells, including cytoskeletal rearrangements [7] and alteration of membrane composition [8]. However, it is still not entirely clear whether a hypoxic environment affects internalization of invasive bacteria such as *Yersinia enterocolitica* into epithelial cells.

*Y*. *enterocolitica* is a gram-negative, facultative intracellular zoonotic pathogen that infects the gastrointestinal tract, causing a variety of diseases like gastroenteritis, acute enteritis and enterocolitis especially in children [9]. The most common source of human infections with *Y*. *enterocolitica* is ingestion of contaminated food [10]. After ingestion, *Y*. *enterocolitica* transverses the intestinal lumen and overlying mucosal layer, across the intestinal epithelial barrier and colonizes the underlying lymphoid tissues [9,11]. The preferential entry of *Y*. *enterocolitica* into ileal Peyer’s patches seems to be facilitated by attachment to and penetration of epithelial microfold (M) cells [12–14]. The uptake by epithelial cells is predominantly mediated by invasin of *Y*. *pseudotuberculosis* [15,16] and *Y*. *enterocolitica* [17,18], but other adhesins like Ail and YadA can contribute to this process [19]. Invasin-promoted internalization is characterized by a “zipper” mechanism [20]. Invasin interacts with high affinity with several members of the β_1_ integrin family through its extracellular C-terminal region [21]. Interaction of invasin of *Y*. *pseudotuberculosis* was shown to bind with a 100 fold higher affinity than the integrin’s natural ligand, fibronectin [22]. Integrins are a family of large transmembrane glycoproteins that function as receptors on the surface of cells, existing as heterodimers of one α and one β subunit, which are non-covalently linked [23]. Among the 18 α and 8 β subunits, β1 integrins are the most widespread [24]. They can be activated by internal as well as external cues, and thus are able to promote inside-out and outside-in signal transduction cascades [25]. Several β1 chain integrins, mainly α5β1 along with α3β1, α4β1, α6β1 and αvβ1, were shown to be receptors for invasin [21]. Invasin binding to integrins triggers receptor clustering, a step that is required for *Y*. *pseudotuberculosis* uptake into host cells [26]. Consequently, a series of signaling cues is initiated, promoting the recruitment of tyrosine kinases like the focal adhesion kinase (FAK) and the involvement of the GTPase Rac1 that induces bacterial entry into non-phagocytic cells [27,28].

The goal of this study is to investigate the effect of hypoxia on the β1 integrin-mediated internalization of *Y*. *enterocolitica* using Caco-2 cells as a polarized intestinal epithelial cell model. We suggest that cellular changes induced by hypoxia lead to a reduction in cell surface localization of host β_1_ integrins thus decreasing invasin-integrin-mediated internalization of *Y*. *enterocolitica* into intestinal epithelial cells.

## Materials and Methods

### Cell culture, bacterial strains and growth conditions

Ethics approval was not required since a commercially available human epithelial colorectal adenocarcinoma, Caco-2, cell line (ATCC® HTB-37™) [29] was used in the project. Cells were maintained in high glucose (4.5 g/L) Dulbecco’s modified Eagle medium (DMEM, Sigma), supplemented with 10% heat-inactivated fetal calf serum (FCS, Gibco BRL), and 50 U/ml Penicillin and 50 μg/ml Streptomycin (Sigma, Germany). Caco-2 cells were grown on polystyrene 24 well plates (Sardstedt, Nümbrecht, Germany) for 6 days post confluency.

The bacterial strains used in this study are *Y*. *enterocolitica* 8081v bioserotype 1A/O:8, patient isolate, wild-type [30] Y1/07 bioserotype 4/O:3, patient isolate, wild-type and YE21 (Y1, **Δ***invA*, kanamycin resistant Kn^R^) [31]. Overnight cultures of *Y*. *enterocolitica* 8081v were grown at 27°C, and *Y*. *enterocolitica* Y1/07 and YE21 were grown at 37°C in Luria-Bertani (LB) broth. The antibiotics used for YE21 selection were carbenicillin 100 mg/ml and kanamycin 50 mg/ml.

Normoxic incubations were performed in a tissue culture incubator at 37°C, 5% CO_2_ in water saturated air, while hypoxic incubations were performed in an oxygen control hypoxia glove box (Coy Laboratory Products, Grass Lake MI, USA) at 37°C, 1% O_2_ and 5% CO_2_ in a humidified (100%) incubation chamber within the glove box. Alternatively, the prolyl-4-hydroxylase inhibitor dimethyloxalylglycine (DMOG; Sigma, Germany) was used to chemically stabilize the HIF-1α subunit under normoxia. DMOG, dissolved in water, was added to the media at 450 μM for 7 hrs.

### Oxygen measurements

Immobilized PSt3 oxygen sensor spots (PreSens, Regensburg, Germany) were attached to the inside of 24 well plates and a polymer optical fiber (POF) was connected to a fiber optic oxygen transmitter that relayed the emitted light to a Fibox4 microprocessor (PreSens, Regensburg, Germany). In this manner, oxygen was measured non-invasively and was not consumed during the process of measurement. Caco-2 cells were grown for 6 days under normoxia and then either moved to 1% O_2_ for 24 hr or kept at normoxia. On day 7 post confluency the cells were infected with *Y*. *enterocolitica* O:8 8081v (see below) and dissolved oxygen was measured at time of infection (24 hr), 1.5, 2.5, 4 and 6 hrs post infection.

### Infection and internalization

Caco-2 cells were seeded at 0.82 x 10^4^ cells/cm^2^ in a 24-well plate with growth area of 1.82 cm^2^ and grown for 6 days post confluence in a normal tissue culture incubator. On day 6, media was changed and two plates were placed at 1% O_2_ for 24 hr while one plate was left under normoxia for 24 hr. One well of Caco-2 cells was counted and cells were used at 1.65–2.75 x 10^6^ cells/cm^2^. Cells were washed three times with PBS and incubated in DMEM without FCS or antibiotics. *Y*. *enterocolitica* O:8 8081v or O:3 Y1/07 strains were grown till OD_600_ = 0.5 and used to infect Caco-2 cells at MOI 10. Plates were centrifuged at 142 *g* for 5 minutes (min) at 20°C and then incubated for 90 min at 37°C at normoxia or hypoxia, accordingly. After 90 min, media was removed and cells were washed with PBS to remove non-associated bacteria. FCS and antibiotic-free media with gentamicin 100 ***μ***g/ml (Sigma, Germany) was added to half of the wells for 60 min and the other half was kept with media only. The supernatant of cells incubated with bacteria and gentamicin was plated to ensure bacterial killing, and also taken for cytotoxicity assays. Cells were washed with PBS to remove antibiotics, trypsinized for 2 min with Trypsin-EDTA (Sigma, Germany), and then lysed with 0.1% Triton X-100 in media. Cell lysates were serially diluted and plated on LB agar. The total number of associated bacteria was determined by counting the colony-forming units (CFU) from wells without gentamicin and the number of internalized bacteria was determined by counting the CFU from wells with gentamicin. Internalization was calculated as percentage of gentamicin surviving bacteria relative to the total number of associated bacteria.

### Blocking of β1 integrin function

Caco-2 cells were grown for 6 days post confluence in a normal tissue culture incubator. On day 6, media was changed and plates were placed at 1% O_2_ for 24 hr while one plate was left under normoxia for 24 hr. One hr before infection, media was removed and replaced with media containing 45 ***μ***g/ml of either 6S6 anti-β1 integrin (Merck-Millipore, Germany) or the mouse IgG1 (Merck-Millipore, Germany). Cells were incubated for 1 hr at 37°C at normoxia or hypoxia. Cells were then washed and infection proceeded as described above using *Y*. *enterocolitica* O8 8081v at MOI 10.

### Infected cell lysis and FAK Western blots

Cells were infected as mentioned in the previous section (infection and internalization) and after 1.5 hrs of infection the media was removed and each well was washed twice with phosphate-buffered saline. Cells were lysed with 1% Triton X-100, 2 mM sodium fluoride, 1 mM ethylenediaminetetraacetic acid in phosphate-buffered saline with protease inhibitor mix (Antipain dihydrochloride 1.48 μM, Pepstatin A 1.46 μM, Leupeptin 10.51 μM, Aprotinin 0.768 μM, Trypsin inhibitors 50 μg/ml and phenylmethanesulfonyl fluoride (PMSF) 1 mM; Sigma, Germany). Subsequently, cells were centrifuged at 17, 000 g at 4°C for 10 min and supernatants including cellular proteins were collected and frozen at -20°C until usage. Equal protein amounts (50 μg) of total cell lysates from each sample were denatured in boiling Laemmli buffer plus 50 mM dithiothreitol for 5 min. Samples were then subjected to 8% sodiumdodecyl sulfate polyacrylamide gel electrophoresis and transferred onto a PVDF membrane (Roth, Germany). Total amount of FAK was detected using FAK (D2R2E) rabbit monoclonal antibody and phosphorylated FAK at Tyr397 was detected using P-FAK Y397 (D20B1) rabbit monoclonal antibody (Cell Signaling Technology, Danvers, MA, USA). β-Actin (Santa Cruz Biotechnology, CA, USA) served as loading control. Quantification of band intensities was performed using Image J 1.48v (National Institutes of Health, USA).

### Whole cell lysis and HIF-1α Western blots

Whole-cell extracts were obtained from Caco-2 cells grown for 6 days post confluency under normoxia. At day 6, they were either left under normoxia or placed under hypoxia for 24 hr after which they were lysed. Supernatants were removed and cells were washed in cold PBS over ice and scraped into 1 ml of lysis buffer (0.1% Nonidet P40, 300 mM NaCl, 10 mM Tris pH 7.9, 1 mM ethylenediaminetetraacetic acid in phosphate-buffered saline), with protease inhibitor mix (Antipain dihydrochloride 1.48 μM, Pepstatin A 1.46 μM, Leupeptin 10.51 μM, Aprotinin 0.768 μM, Trypsin inhibitors 50 μg/ml and phenylmethanesulfonyl fluoride (PMSF) 1 mM; Sigma, Germany). Subsequently, cells were centrifuged at 17, 000 *g* at 4°C for 10 min and supernatants including cellular proteins were collected and frozen at -20°C until use. Equal protein amounts (50 μg) of total cell lysates from each sample were denatured in boiling Laemmli buffer plus 50 mM dithiothreitol for 5 min. Samples were then subjected to 8% sodiumdodecyl sulfate polyacrylamide gel electrophoresis and transferred onto a PVDF membrane (Roth, Germany). β1 integrin was detected with a purified mouse anti-Integrin β1 antibody (BD Transduction Laboratories, USA). HIF-1 α was detected with a purified rabbit anti-human HIF-1α antibody (Merck-Millipore, Temecula, CA, USA). β-Actin (Santa Cruz Biotechnology, CA, USA) served as loading control. Quantification of band intensities was performed using Image J 1.48v (National Institutes of Health, USA).

### Brush border membrane isolation and sucrase activity

Caco-2 cells grown for 6 days post-confluency under normoxia. At day 6, they were either left under normoxia or placed under hypoxia for 24 hr. Brush border membranes of Caco-2 cells were isolated by the divalent cation precipitation method [32,33]. Cells were homogenized using a Potter–Elvehjem homogenizer in the hypertonic homogenization buffer (300 mM Mannitol, 12 mM Tris-HCl pH 7.1) supplemented with protease inhibitor mix (Antipain dihydrochloride 1.48 μM, Pepstatin A 1.46 μM, Leupeptin 10.51 μM, Aprotinin 0.768 μM, Trypsin inhibitors 50 μg/ml and phenylmethanesulfonyl fluoride (PMSF) 1 mM; Sigma, Germany). The homogenates were passed through a Luer-21 Gage needle and CaCl_2_ was added to a final concentration of 10 mM and then centrifuged at 5,000 x *g* for 15 min to obtain the homogenate fraction (H). Homogenates were then incubated at 4°C for 30 min with gentle agitation and centrifuged again at 5,000 x *g* for 15 min. The pellet was then resuspended in 10 mM Tris-HCl + 150 mM NaCl pH 7.4 to obtain the basolateral and microsomal membrane vesicle fraction (P1). The supernatant was centrifuged at 25,000 x *g* for 30 min and the pellet was resuspended in 10 mM Tris-HCl + 150 mM NaCl pH 7.4 to yield the apical membrane/brush border membrane fraction (P2) while the supernatant contained all other soluble and small vesicular membrane-bound fraction (S).

Subsequently, 50 μg of total cell lysates from each sample were denatured in boiling Laemmli buffer plus 50 mM dithiothreitol for 5 min. Samples were then subjected to 8% sodiumdodecyl sulfate polyacrylamide gel electrophoresis and transferred onto a PVDF membrane (Roth, Germany). β1 integrins were detected with a purified mouse anti-Integrin β1 antibody (BD Transduction Laboratories, USA) and sucrase isomaltase was detected using mAb anti-SI antibody HBB 3/705 [33] obtained from Drs. Hans-Peter Hauri and Erwin Sterchi (University of Basel and University of Bern, Switzerland).

Sucrase activity in the homogenates, basolateral membranes (P1 fraction), supernatant (S fraction) and brush border membranes (P2 fraction) was measured using 150 mM sucrose added to 25 μl of sample and end glucose was detected using GOD PAP fluid (Axiom Diagnostics, Worms, Germany) at 492 nm. Sucrase specific activity was calculated as μM.hour^-1^.mg^-1^ of protein.

### Immunofluorescence

Caco-2 cells were grown on glass on cover slips in a 24-well plate for 6 days post confluence in a normal tissue culture incubator. On day 6, media was changed and plates were placed at 1% O_2_ or left under normoxia for 24 hr. Cells were fixed with ice-cold methanol for 15 min and washed with Tris buffered saline with 0.01% Tween 20 (TBS-T). Coverslips were then incubated in blocking solution of 3% BSA with 0.01% TBS-T for 30 min at room temperature followed by permeabilization using 0.3% Triton X-100 for 15 min at room temperature. After washing, coverslips were incubated with 0.01 mg/ml mouse anti-β1 integrin (Merck-Millipore, Germany) or the mouse IgG1 isotype control (Merck-Millipore, Germany) diluted in 3% BSA with 0.01% TBS-T at room temperature for 2 hr. Coverslips were washed with 0.01% TBS-T and incubated with secondary goat anti-mouse Alexa Fluor® 488-labeled antibody (Invitrogen, Germany) for 45 min at room temperature, protected from light. After washing, coverslips were embedded in ProlongGold + DAPI™ (Invitrogen, Germany). Microscopy was performed using a Leica TCS SP5 confocal fluorescence microscope with a HCX PL APO 40X 0.75–1.25 oil immersion objective. Gain settings were kept the same when acquiring images of cells grown under the two conditions.

### Statistical analysis

All experiments were performed in duplicate three independent times. Data were analyzed using Excel 2010 (Microsoft) and GraphPad Prism 6.0 (GraphPad Software). Differences between two or more groups were analyzed by using a One-way ANOVA with Tukey's multiple comparisons test. For Western blots and DMOG internalization statistics, unpaired, two-tailed Student’s *t*-tests were performed. The significance is indicated as follows: ns = non-significant, * p ≤ 0.05; ** p ≤ 0.01, *** p ≤ 0.001 and **** p < 0.0001.

## Results

### Characterization of oxygen conditions during *Y*. *enterocolitica* invasion into Caco-2 cells

**Fig 1:**
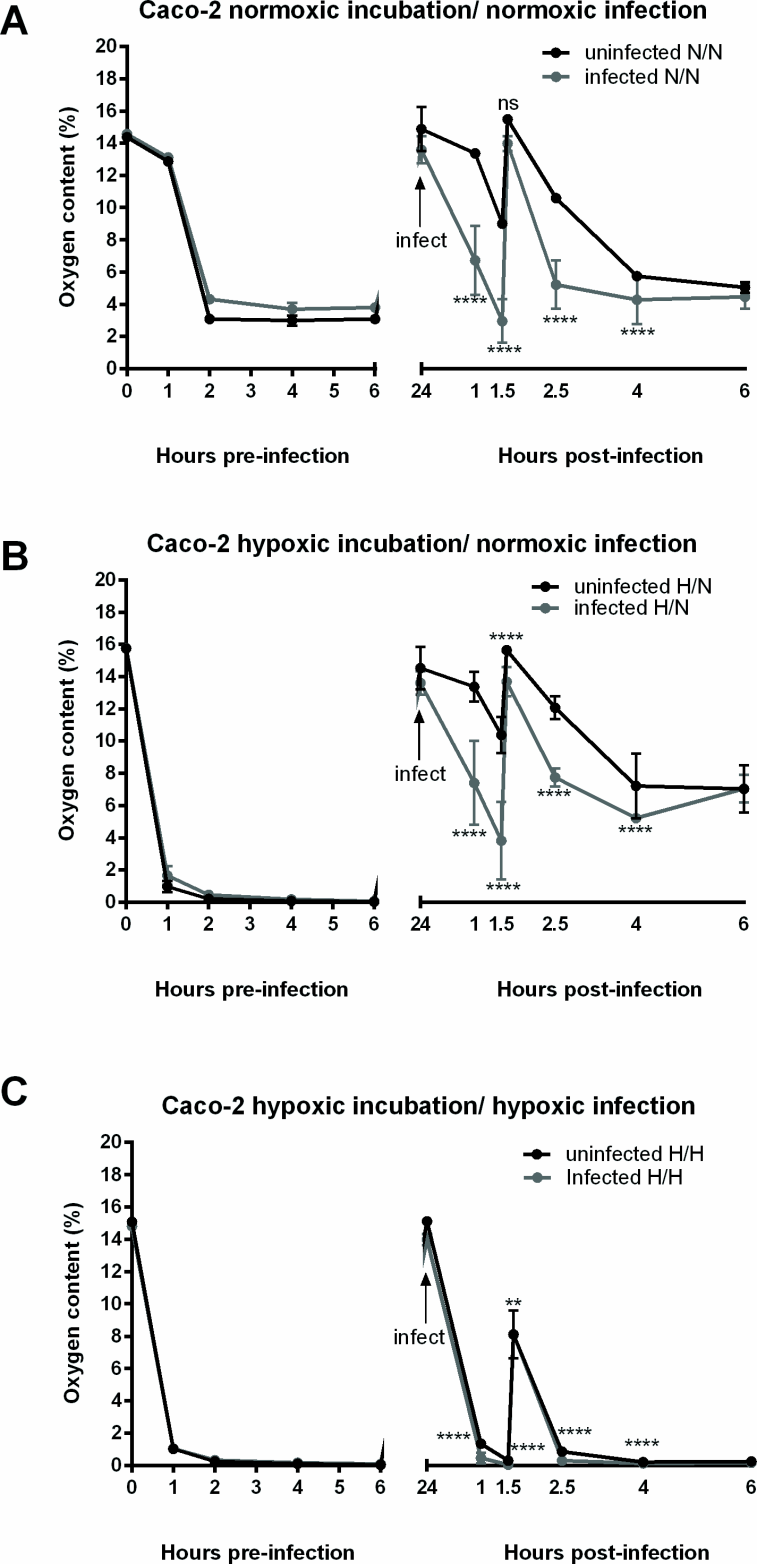
Oxygen levels in Caco-2 cultures. Caco-2 cells were grown for 6 days post confluence and then placed under hypoxia or kept under normoxia. (A) Measurements in normoxic pre-incubated and normoxic infected (or uninfected) cells, (B) measurements in hypoxic pre-incubated and normoxic infected (or uninfected) cells and (C) measurements in hypoxic pre-incubated and hypoxic infected (or uninfected) cells. Oxygen peaks represent the addition of fresh media: at time point 24 hr fresh media with bacteria, and at time point 1.5 hr post infection fresh media with gentamicin. Plotted values represent mean ±SEM and are displayed as % oxygen. ** p **≤** 0.01, **** p < 0.0001and ns = non-significant using two-tailed Student’s *t*-test.

In order to study the host pathogen interactions under hypoxia, the experimental settings of the culture conditions needed to be established. For our purposes, we used Caco-2 cells. This human cell line was grown to a monolayer with differentiated polarized intestinal epithelial cells [34]. Differentiated Caco-2 cells develop brush-border microvilli typical of intestinal enterocytes and express a multitude of intestinal enzymes like sucrase-isomaltase [34,35]. Interestingly, it has been recently shown that in Caco-2 polarized epithelial cell lines, β_1_ integrins can be found apically at the tight junctions, colocalizing with the zonula occludens proteins [36]. Furthermore, dissolved oxygen levels in the cell culture media were measured using optical sensors, based on the oxygen-dependent quenching of phosphorescent probes that is proportional to the oxygen level in the immediate surroundings [2,37]. Infection incubations were performed under normoxia or hypoxia, thus resulting in three distinct conditions: normoxic pre-incubation / normoxic infection, hypoxic pre-incubation / normoxic infection and hypoxic pre-incubation / hypoxic infection. Oxygen measurements were performed over the course of 6 hours (hr) before infection and 6 hr following infection with *Y*. *enterocolitica* 8081v with an MOI of 10 (see experimental procedures for details). Normoxic pre-incubation of uninfected cells resulted in oxygen levels lower than 4% after 6 hr (Fig 1A, left panel). After normoxic infection at time point 24 hr, cells show oxygen levels that decreased much faster than uninfected cells before similar levels (5% O_2_) are reached after 6 hr (post infection) (Fig 1A, right panel). Hypoxic pre-incubated cells reach levels of approximately 0.04% O_2_ after 6 hr (Fig 1B and 1C, left panels). Hypoxic pre-incubated cells that were infected under normoxia show a faster decrease in oxygen levels as compared to uninfected cells and finally reach 7% O_2_ after 6 hr post infection (Fig 1B, right panel). Hypoxic pre-incubated cells that were infected under hypoxia also show a slight yet significant difference in oxygen levels as compared to uninfected cells and finally reach 0.2% O_2_ after 6 hr of infection (Fig 1C, right panel). It is important to note that after 1.5 hrs of infection in all culture conditions, fresh media with or without gentamicin was added to the cells and corresponds to the peak in oxygen levels that immediately follow.

### Hypoxic pre-incubation reduces *Y*. *enterocolitica* internalization

**Fig 2:**
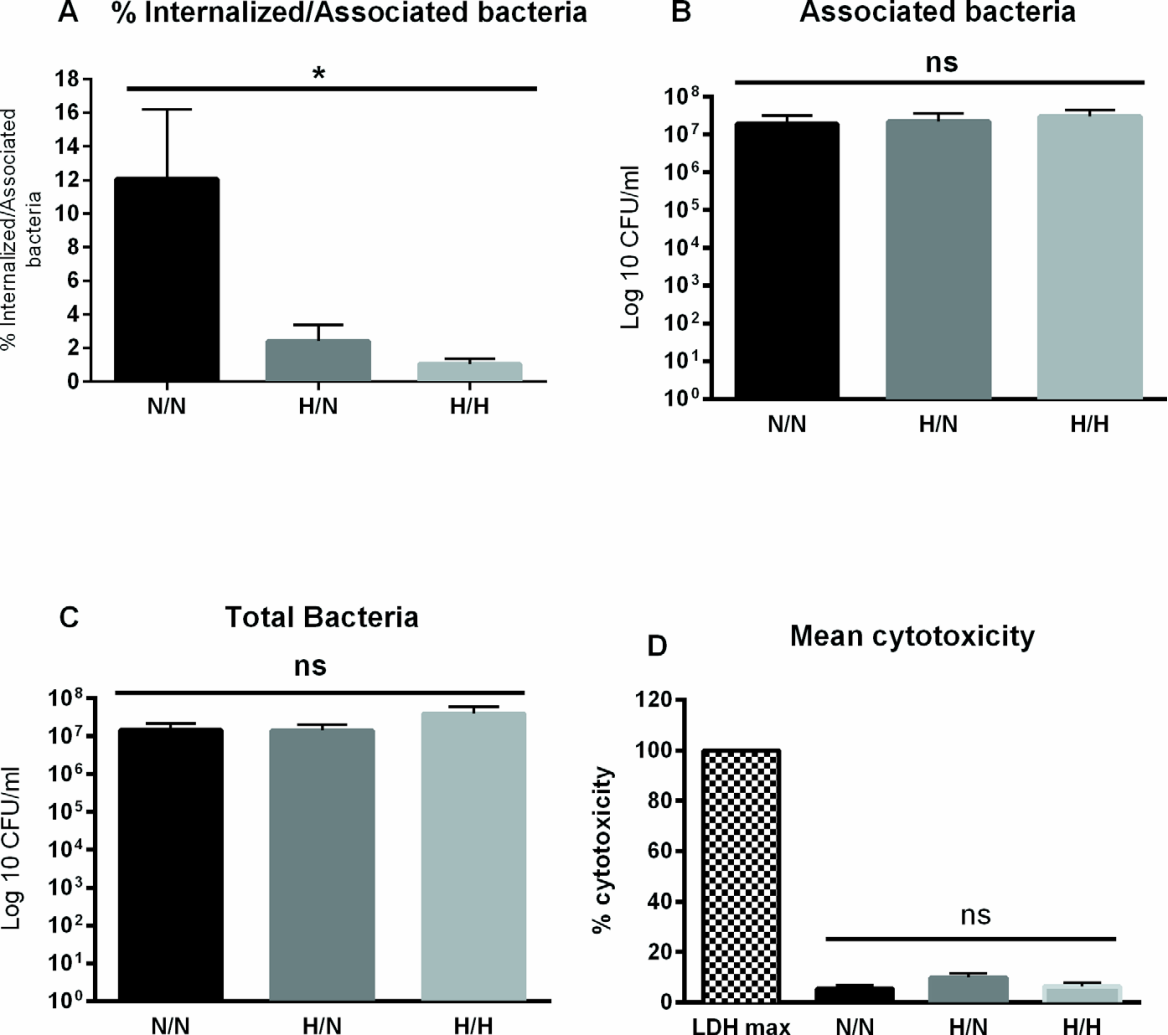
*Y*. *enterocolitica* internalization is reduced in hypoxic incubated cells. *Y*. *enterocolitica* serotype O:8 8081v was used to infect Caco-2 cells (MOI 10) pre-incubated at normoxia or hypoxia for 24 hr. The infection was also performed at normoxia or hypoxia. (A) The percentage of internalized bacteria was significantly reduced in hypoxia pre-incubated cells. There was no significant difference in the number of associated bacteria (B) or in bacterial growth (C) in the cells grown under either condition. (D) Twenty-four hr incubation under hypoxia did not result in significant differences in cytotoxicity as compared to 24 hr under normoxia. * p ≤ 0.05 using one-way ANOVA. Plotted values represent mean ±SEM.

Caco-2 cells were grown for 6 days under normoxia and then either moved to 1% O_2_ for 24 hr or kept at normoxia. After addition of *Y*. *enterocolitica* O:8 8081v at a multiplicity of infection (MOI) 10, plates were centrifuged in order to obtain uniform bacterial attachment to host cells and numbers of intracellular bacteria were identified by gentamicin survival assay [38]. Fig 2A shows that cells pre-incubated under hypoxia had a significantly decreased number of internalized bacteria, after normoxic and hypoxic infection, compared to the normoxic control. Normoxic Caco-2 showed 12% internalized bacteria while hypoxic pre-incubated cells showed 2.4 and 1% internalized bacteria during normoxic and hypoxic infections respectively. Fig 2 shows that there was no significant difference in either the number of associated bacteria (2 B) or in the total bacterial number (2 C) respectively, in the different oxygen incubations. Finally, a lactate dehydrogenase assay (LDH) confirmed no significant cytotoxic effect of hypoxic incubation of Caco-2 cells (Fig 2D).

### Beta one (β_1_) integrin-mediated internalization

**Fig 3:**
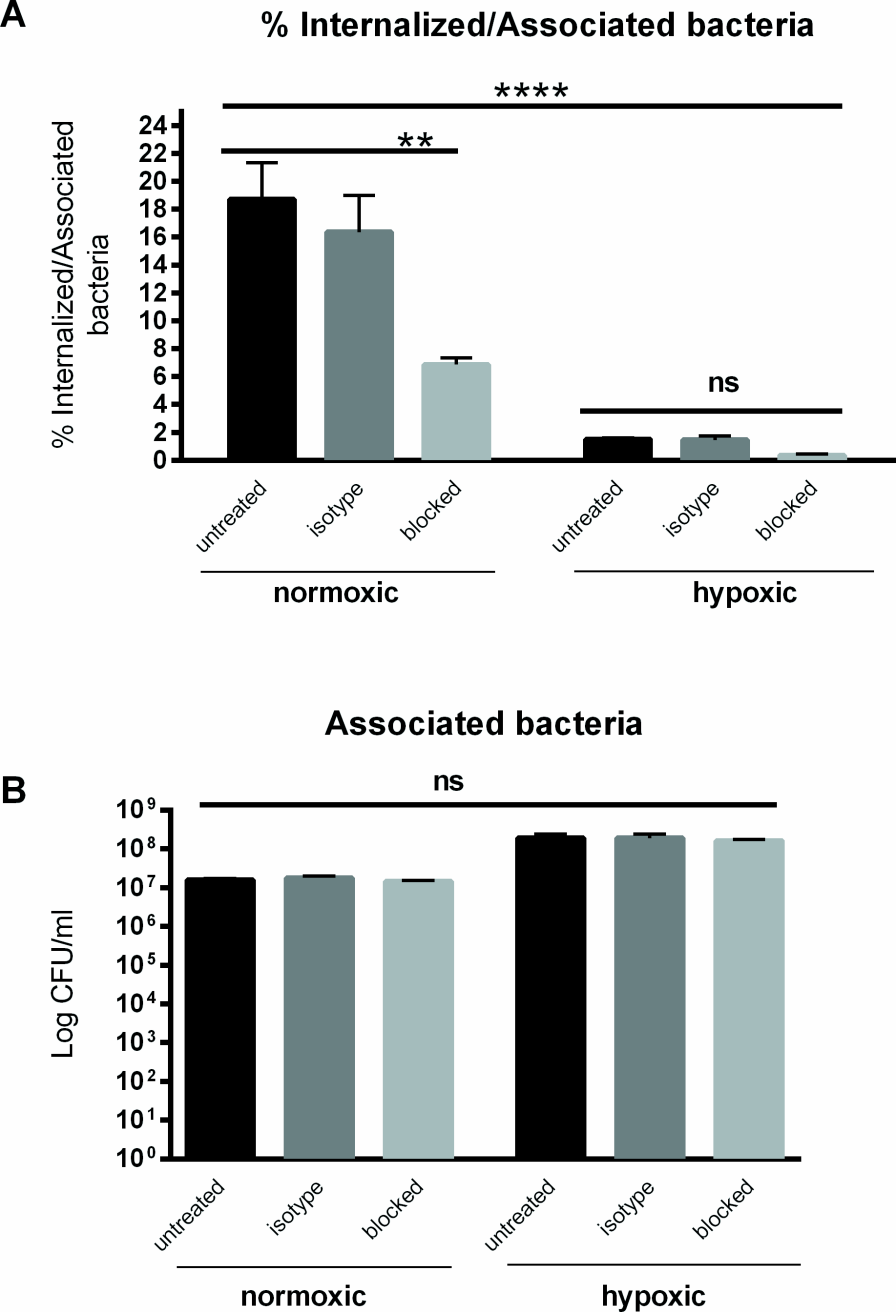
Integrin blocking decreases internalization. Cells were treated with 45 μg/ml of 6S6 integrin blocking antibody, 45 μg/ml of IgG1 isotype control or left untreated for one hour before infection. (A) The percentage of internalized bacteria in cells blocked with anti-integrin antibody was significantly decreased. There was no significant difference between untreated or antibody blocked cells under hypoxia. (B) There was no significant difference in the number of associated bacteria under any condition. **** p<0.0001 using One way ANOVA test, and ** p ≤ 0.01 and ns = non-significant using Tukey's multiple comparisons test.

In order to confirm the role of host β_1_ integrins in *Yersinia enterocolitica* entry into intestinal epithelial cells, β_1_ integrins were functionally blocked by using a 6S6 anti-β1 integrin antibody that binds to the extracellular fragment of the receptor. The normoxic or hypoxic incubated Caco-2 cells were treated for 1 hour and were then infected with *Y*. *enterocolitica* O:8 8081v at MOI 10. The results in Fig 3A show a significant decrease in bacterial internalization in β_1_-integrin-blocked cells as compared to the controls under normoxia. Percent internalization was 6.8% for blocked as compared to 18.7% in untreated cells and 16% in IgG1 isotype-treated cells, in line with previous blocking studies [21]. Blocking of β_1_ integrins under hypoxia resulted in a slight but not significant decrease, 0.3% for blocked compared to 1.5 and 1.4% in untreated and isotype control cells, respectively (Fig 3A). In summary, blocking under hypoxia revealed a strong decrease in internalization when compared to blocking under normoxia, whereas the number of associated bacteria was comparable between the blocked and untreated controls under either oxygen condition (Fig 3B).

### Invasin-mediated internalization

**Fig 4:**
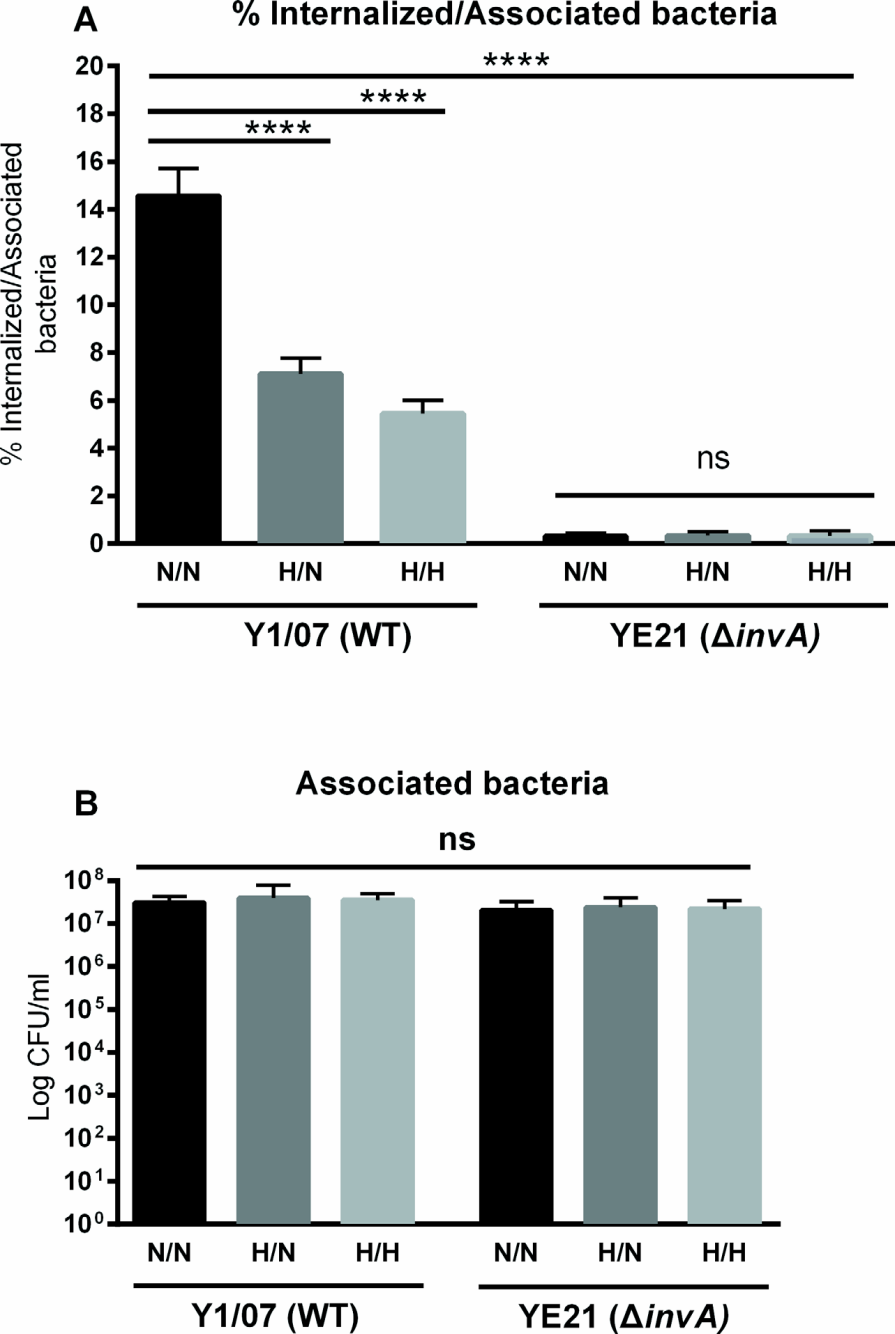
*Yersinia* invasin is required for internalization. *Y*. *enterocolitica* serotype O:3 Y1/07 (MOI 10) and the isogenic *inv* deletion mutant YE21 were used to infect Caco-2 cells pre-incubated at normoxia or hypoxia for 24 hrs. The infection was also performed at normoxia or hypoxia. (A) The percentage of internalized wild type bacteria was significantly reduced in hypoxia pre-incubated cells. Infection with the invasin-deficient strain YE21 showed no significant difference between the different oxygen conditions. (B) There was no significant difference in the number of associated bacteria in the cells grown under either condition. **** p < 0.0001 using One way ANOVA test, and **** p < 0.0001 and ns = non-significant using Tukey's multiple comparisons test.

In order to investigate the invasin-β_1_ integrin mediated internalization, two strains of *Y*. *enterocolitica* serotype O:3 were used to infect normoxic and hypoxic incubated Caco-2 cells. The *Y*. *enterocolitica* strain Y1/07 is a wild type invasin-expressing strain while YE21 is the respective invasin-deficient mutant strain (Δ*invA)* [31]. Similarly to the infection with 8081v, 6-day post confluent Caco-2 cells were pre-incubated either at normoxia or hypoxia for 24 hr. Infection incubations were also performed under normoxia or hypoxia. The results in Fig 4A show that the wild type strain was internalized significantly less in cells pre-incubated under hypoxia, similar to the O:8 serotype. Infection with the invasin mutant showed a highly significant decrease in internalization as compared to the infection with the wild type strain (Fig 4A). Similar to the *Y*. *enterocolitica* O:8 8081v wildtype strain, the number of associated bacteria showed no significant difference between the different oxygen conditions for either bacterial strain (Fig 4B).

### Influence of oxygen levels on FAK activation

**Fig 5:**
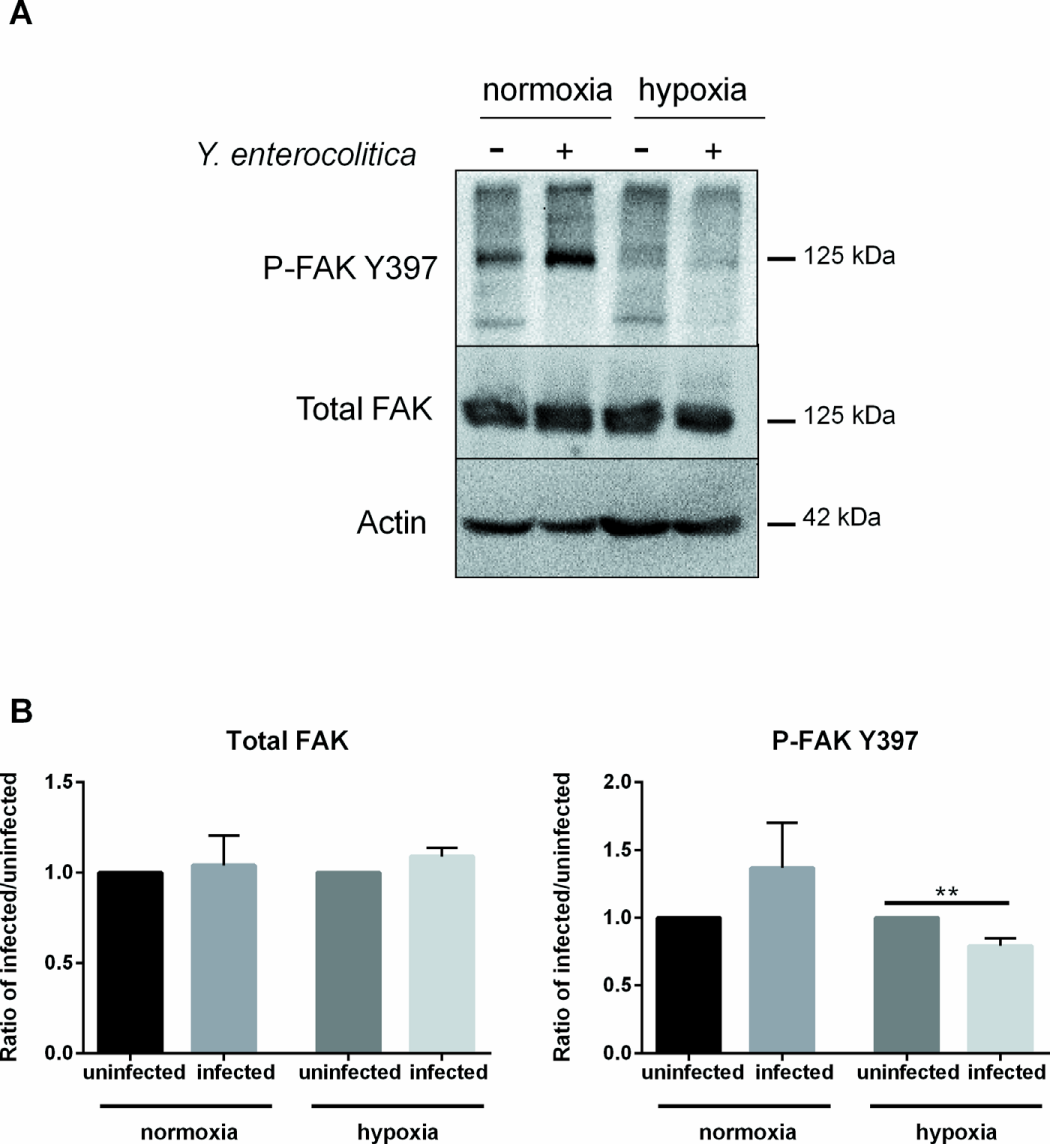
Analysis of total and phosphorylated FAK. Levels of total FAK and p-FAK Y397 from Caco-2 cells pre-incubated under hypoxia or normoxia for 24 hr and then infected with *Y*. *enterocolitica*. (A) Western blots and (B) their quantification. Values are presented as a ratio of infected over uninfected in case of normoxia or hypoxia, respectively. ** p ≤ 0.01 using two-tailed Student’s *t*-test.

The efficient uptake of *Yersinia* spp. into non-phagocytic cells via the invasin-integrin pathway requires the FAK that plays a central role in downstream signaling events [39]. Binding of integrin leads to an increase in tyrosine phosphorylation levels in the cell, specifically at tyrosine 397, identified as the major site of autophosphorylation in cell adhesion [40,41]. Therefore, in order to determine whether the hypoxia-induced decrease in internalization is also correlating with altered levels of phosphorylated FAK (p-FAK), total and p-FAK level were analyzed in cells that were pre-incubated under normoxia or hypoxia and then infected with *Y*. *enterocolitica*. Western blots show an increase in p-FAK at the site Y397 after infection as compared to uninfected cells under normoxia (Fig 5A). Under hypoxia, the levels of p-FAK are low in uninfected cells, and were further reduced upon infection (Fig 5A). Quantification of the Western blots shows that when compared to uninfected cells, infected cells show a distinct but not significant increase in p-FAK under normoxia and a significant decrease in p-FAK under hypoxia (Fig 5B). Total FAK levels showed no overall difference between infected and uninfected cells under either oxygen condition (Fig 5B).

### Beta one (β_1_) integrin and HIF-1 alpha (α) protein levels

**Fig 6:**
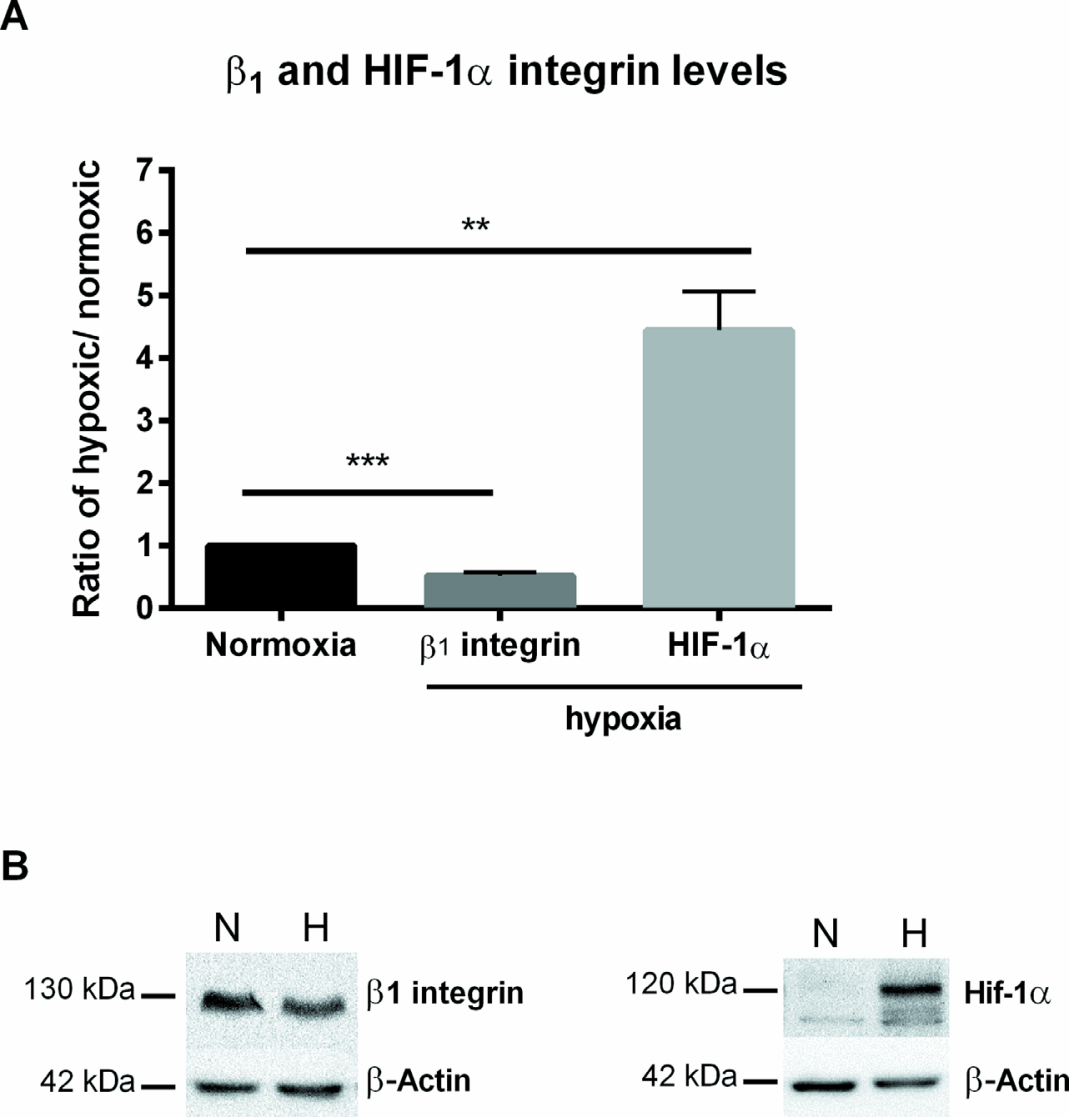
Quantification of Western blots of β1 integrin and HIF-1α. (A) Caco-2 cells pre-incubated under hypoxia as compared to the normoxic controls for 24 hr. Quantification of Western blot values are presented as a ratio over the respective normoxic value. (B) Representative Western blots showing β_1_ integrin at 130 kDa and HIF-1α at 120kDa. ** p ≤ 0.01, *** p ≤ 0.001 using two-tailed Student’s *t*-test.

The uptake of *Y*. *enterocolitica* into Caco-2 cells requires binding to host β_1_ integrins, and since decreased bacterial entry was seen under hypoxia, it was important to investigate whether reduced oxygen conditions induce changes in β_1_ integrin protein levels. Thus, Western blots were performed on whole cell lysates from 7-day post confluent Caco-2 cells incubated under normoxia or hypoxia for 24 hr. Interestingly, β_1_ integrin protein levels were significantly decreased (0.5-fold) under hypoxia. Lower β_1_ integrin protein levels (Fig 6) may explain the hypoxia-mediated decrease of the *Y*. *enterocolitica* internalization rate. At the same time, protein level of the transcription factor hypoxia inducible factor HIF-1α, a global regulator of cellular response to hypoxia [42] was significantly increased (4-fold) in hypoxic incubated cells (Fig 6).

**Fig 7:**
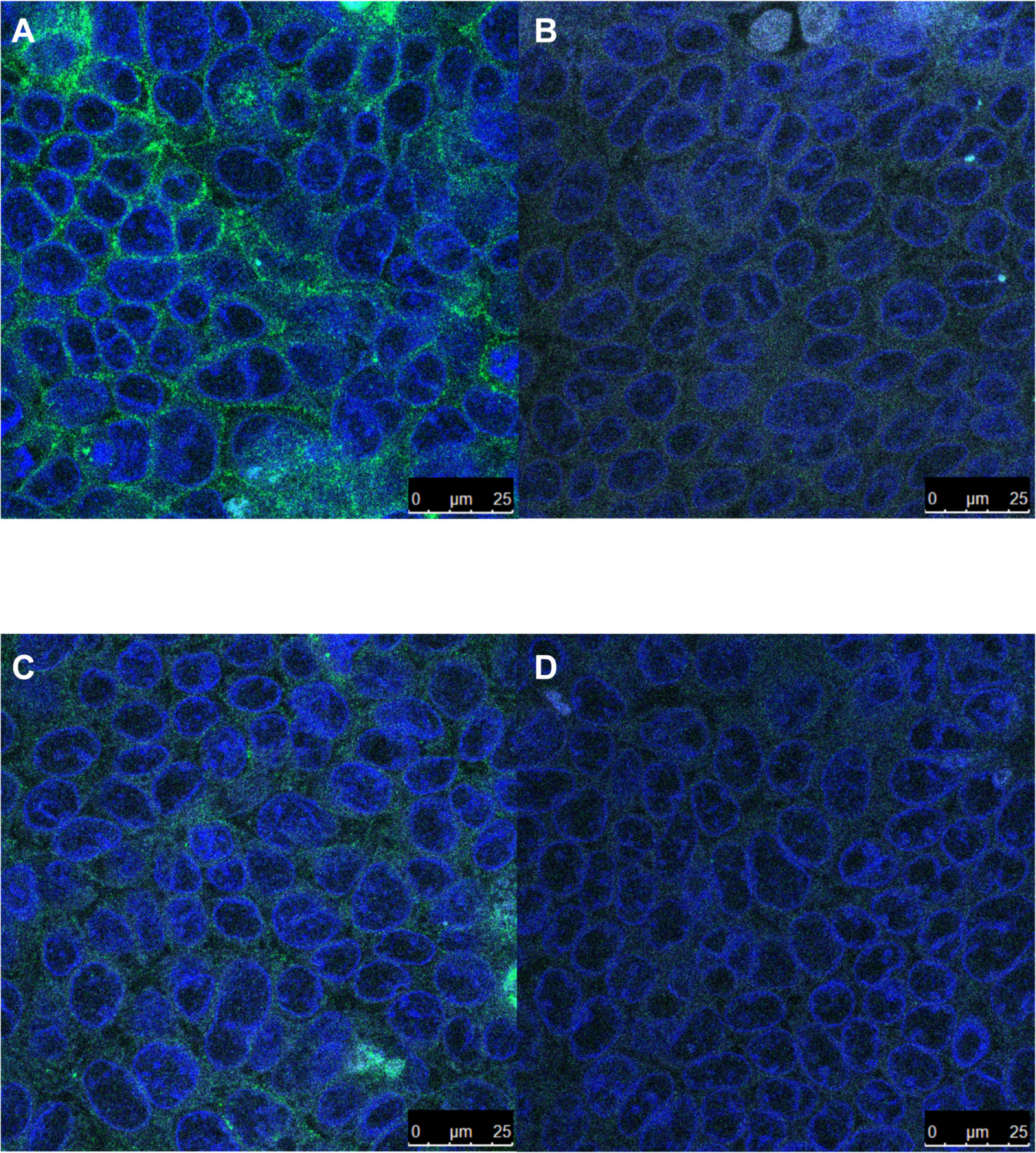
Decreased β1 integrin under hypoxia. Representative fluorescent micrographs of β_1_ integrin abundance under normoxia (A) or hypoxia (C) with the mouse IgG1 isotype control (B and D). Green: β1 integrin, Blue: DAPI.

In order to confirm the decrease in β_1_ integrin protein levels seen in the Western blots, immunofluorescent visualization of β_1_ integrins in 7-day post confluent Caco-2 cells incubated under normoxia or hypoxia for 24 hr was performed. Representative images shown in Fig 7 confirm a distinct decrease in β_1_ integrin intensity, visualized in green, and distribution on the cells in hypoxic samples (Fig 7C) compared to the normoxic controls (Fig 7A). The isotype controls for normoxic and hypoxic staining are shown in Fig 7B and 7D respectively.

### Apical localization of β_1_ integrin

Differentiated Caco-2 cells display well-developed, brush-border membranes and express the active, apically enriched, intestinal disaccharidase sucrase-isomaltase (SI) [34,35]. To monitor any changes in cell polarization as a consequence of hypoxic exposure, the enrichment levels and activity of brush border SI was assessed in hypoxia pre-incubated cells. Furthermore, apical cell surface enrichment of β1 integrin was determined in hypoxic pre-incubated cells and compared to normoxic pre-incubated cells. For this, brush border membranes were separated from intracellular and basolateral membranes by the use of divalent ions (Ca^2+^) and subsequent separation by centrifugation (see material and methods section).

**Fig 8:**
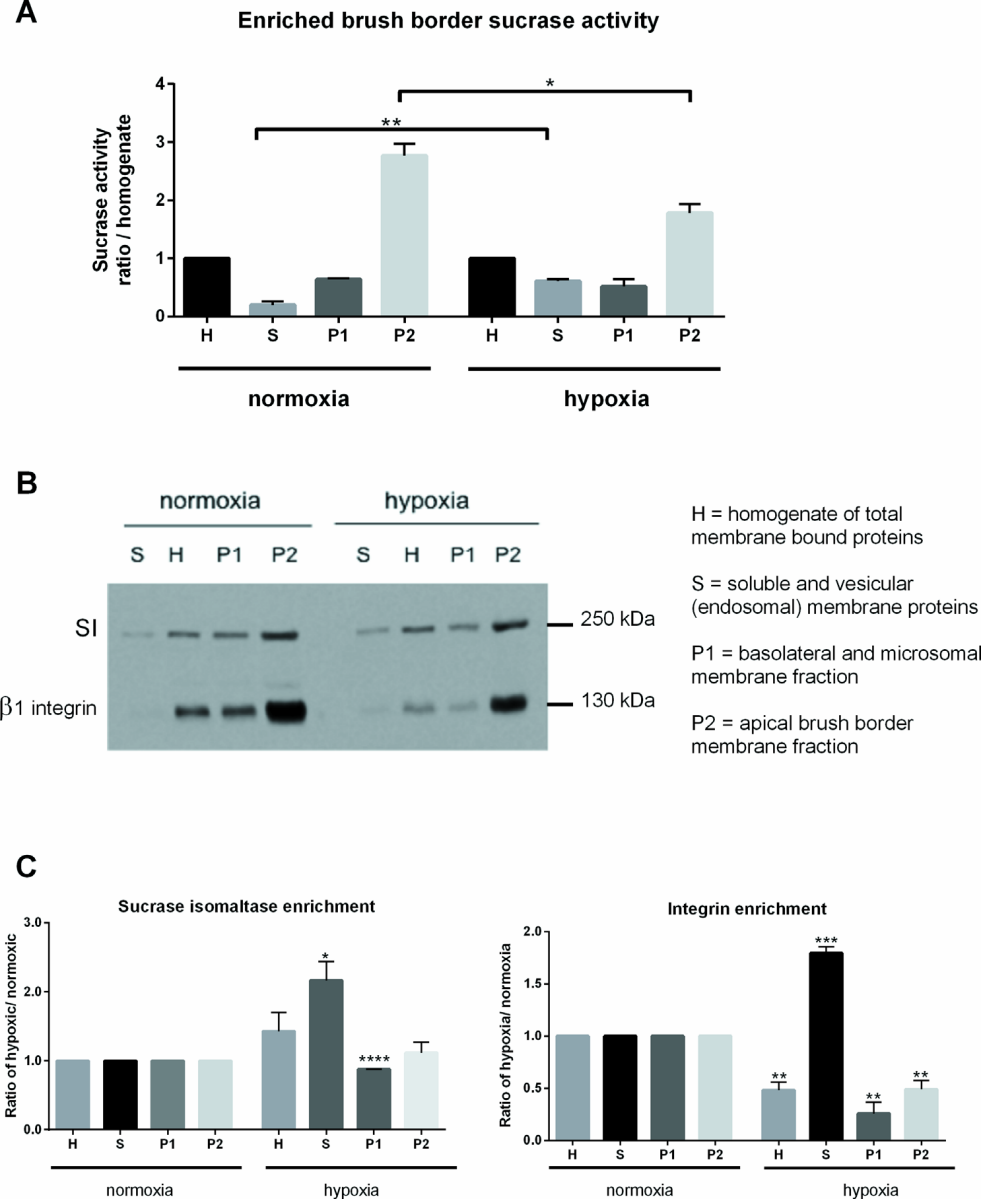
Analysis of brush border membrane protein enrichment. (A) Analysis of sucrase activity in the different membrane fractions under normoxia and hypoxia. (B) Western blots showing the localization of SI and β1 integrin in the different membrane fractions under normoxia and hypoxia. (C) Quantification of SI and β1 integrin enrichment in the different membrane fractions under normoxia and hypoxia. Values are presented as a ratio of each fraction over homogenate. * p ≤ 0.05; ** p ≤ 0.01, *** p ≤ 0.001 and **** p < 0.0001 using two-tailed Student’s *t*-test.

First, sucrase activity was assessed in the brush border (P2) fraction versus the total cellular homogenates (H). As expected for well-differentiated cells, under normoxia, the activity of sucrase in P2 was approximately 3-fold higher than in the homogenate [33]. Interestingly, sucrase activity was significantly lower under hypoxia, with activity of sucrase in P2 less than 2-fold higher than in the homogenate fraction (Fig 8A). Next, the patterns of SI protein enrichment in the different membrane fractions was analyzed by Western blots (Fig 8B). As expected, SI protein bands were mostly enriched in the P2 fraction under normoxia, in good correlation with the specific activity (Fig 8A). Under hypoxia, SI bands were significantly increased in the soluble and homogenate fractions with a slight decrease in P1 (Fig 8C).

Localization of β1 integrin under normoxia was highly enriched in the P2 fraction under normoxia, confirming its apical cell surface localization. Under hypoxia, β1 integrin levels were reduced in the H, P1 and P2 fractions accompanied by a significant increase in soluble vesicular membrane localization compared to normoxia (Fig 8C).

In summary, these results indicate that under normoxia, in 7-day postconfluent Caco-2 cells, β1 integrin receptors are found on the apical surface. In contrast, after 24 hrs of incubation under hypoxia, apical cell surface localization of β1 integrins was significantly decreased, in agreement with the observed decreased bacterial internalization shown above.

### Treatment of Caco-2 cells with DMOG reduces *Y*. *enterocolitica* internalization

**Fig 9:**
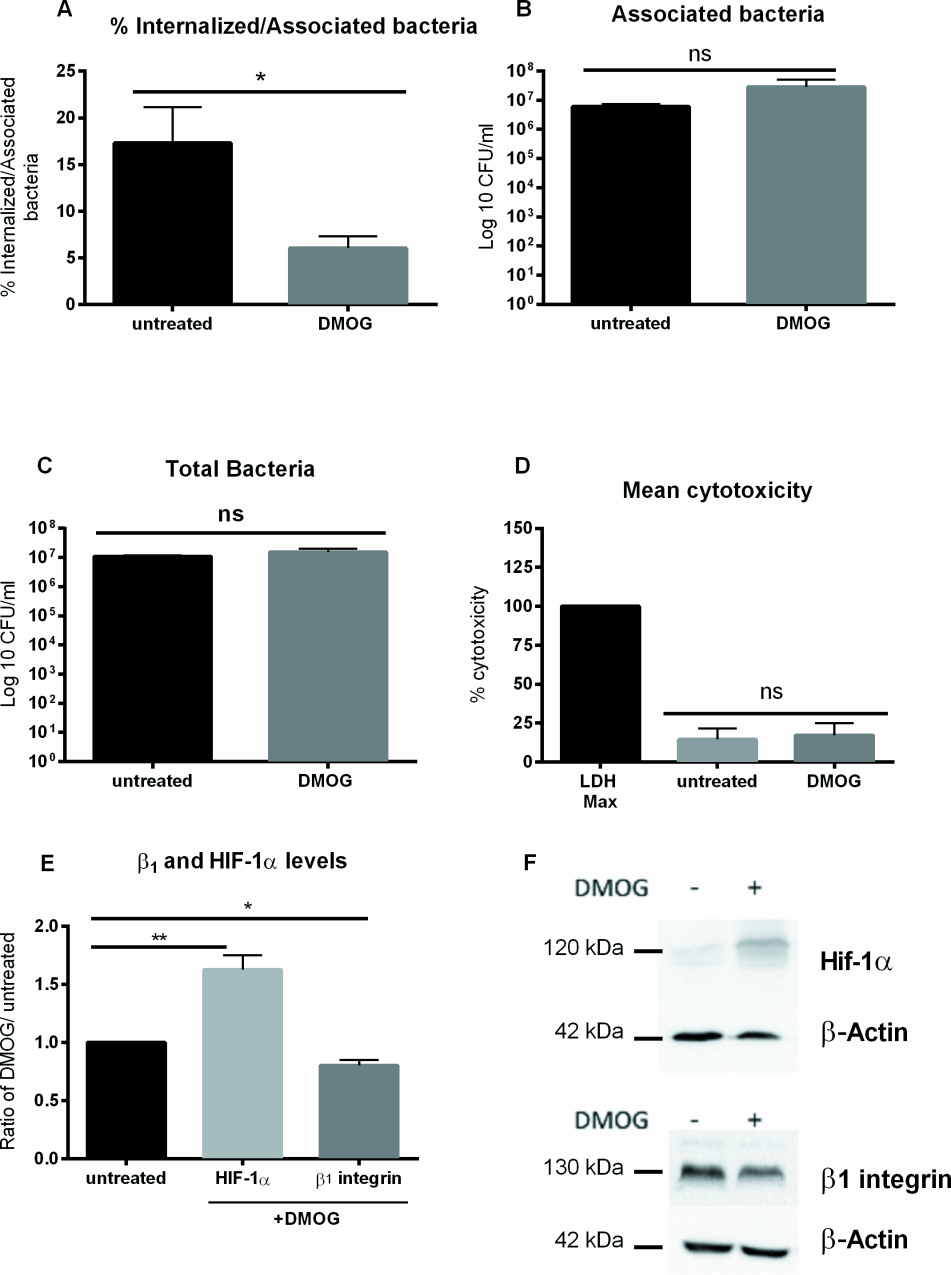
Treatment with DMOG mimics hypoxic results. *Y*. *enterocolitica* serotype O:8 8081v was used to infect Caco-2 cells (MOI 10) incubated at normoxia and treated with DMOG for 7 hr. The infection was performed at normoxia. (A) The percentage of internalized bacteria was significantly reduced in DMOG treated cells. There was no significant difference in the number of associated bacteria (B) or in bacterial growth (C) in the cells between treated and untreated. (D) Treatment with DMOG did not result in significant differences in cytotoxicity. (E) Western blots of β1 integrin and HIF-1α and their (F) quantification in Caco-2 cells treated with DMOG for 7 hr as compared to untreated controls. * p ≤ 0.05; ** p ≤ 0.01 using two-tailed Student’s *t*-test.

In order to determine whether HIF-1α plays a role in *Y*. *enterocolitica* uptake, a pharmacological agent was used to stabilize HIF-1α under normoxia. Dimethyloxalylglycine (DMOG) is a competitive pan inhibitor of prolyl-4-hydroxylases that degrade HIF-1α and it has been effectively used to stabilize HIF-1α in cells under normoxia [43]. Therefore, 7 day post confluent Caco-2 cells were treated with DMOG or with media alone under normoxic conditions. The cells were then infected with *Y*. *enterocolitica* O:8 8081v with a MOI 10 (under normoxia), DMOG was kept in the media of treated cells throughout the infection process. Fig 9A shows a significant decrease in bacterial internalization in cells treated with DMOG (6%) as compared to the untreated control (17.3%). Neither the number of associated bacteria or bacterial growth control showed a significant difference between DMOG treated cells and untreated controls (Fig 9B and 9C, respectively). Cytotoxicity of DMOG on Caco-2 cells was determined by performing an LDH assay, and no significant cytotoxic effect of DMOG treatment was found (Fig 9D). Furthermore, we found that DMOG treatment resulted in a slight, but significant decrease (0.8-fold) in β_1_ integrin and a significant increase (1.6-fold) in HIF-1α protein levels as compared to the untreated control (Fig 9E and 9F). These data imply a possible involvement of HIF-1α in the decreased β_1_ integrin levels that in turn lead to a reduced internalization of *Y*. *enterocolitica*.

## Discussion

The zoonotic bacterium *Yersinia enterocolitica* colonizes the human intestinal epithelium and its uptake is mediated by bacterial invasins that bind to host cell surface β_1_ integrins. In human intestinal enterocytes, β_1_ integrins are mostly localized on the basal and basolateral surfaces, however, on M cells, they are found mostly apically [44,45]. Studies have shown that M cells are the primary site of *in vivo* intestinal epithelial invasion by *Yersinia* species [12–14]. However, since oral *in vivo* infection with *Y*. *enterocolitica* was found to be lethal to mice, a direct correlation to human infections cannot be made [46]. Furthermore, since M cells represent less than 1% of the total human intestinal surface [47,48], it is much more relevant and efficient to study bacterial invasion in polarized epithelial cells models such as CHO, HEp-2, MDCK and Caco-2 [36,49,50]. Caco-2 cells are a well-established model for the intestinal epithelium, due to their ability to polarize and differentiate into intestinal epithelial cells [34]. It has been described that Caco-2 cells exist in three different states in culture: homogeneously undifferentiated at subconfluence, heterogeneously polarized and differentiated between 0 and 20 days after confluence, and homogeneously polarized and differentiated after 30 days [51]. Furthermore, in the intermediate state, all cells exhibit apical cell-cell junctions and polarization but they display a high degree of heterogeneity in the organization of the apical surface and the development of the brush border membrane [52]. In parallel to this differentiation process, a differential pattern of expression of extra cellular matrix (ECM) proteins and integrins along the crypt-to-villus is seen in epithelial cells [53]. While undifferentiated cells express integrins along the entire cell surface, as cells differentiate, the integrins begin to exhibit a shift from a lateral to a more basal distribution [50,54]. Interestingly, a comparative study of monocultures of Caco-2 on plastic plates and co-cultures with human intestinal mesenchymal (HIM) cells revealed a better basal distribution of β1 integrins in the co-culture system [55]. Conversely, β1 integrins in polarized MDCK and Caco-2 cells displayed an apicolateral distribution, where they colocalized with tight junction proteins and enabled *Y*. *pseudotuberculosis* internalization [36]. This disparity may be due to the varying differentiation stages at which the cells were used. Indeed, studies have shown that at advanced stages of cellular differentiation, bacterial internalization frequencies are substantially reduced [50,56,57]. We have shown that in our Caco-2 culture system, 7-day postconfluent cells display an apical cell surface localization of β1 integrins (Fig 8).

This may explain that *Y*. *enterocolitica* uptake can still be detected in differentiated Caco-2 cells in a cell culture model. In this study we show that hypoxic pre-incubated cells show less internalization of *Yersinia enterocolitica* compared to cells kept under normoxia. This phenomenon was in line with decreased protein levels of host β_1_ integrin in hypoxic cells. The results of hypoxic pre-incubation of Caco-2 cells infected with *Y*. *enterocolitica* under normoxia largely excludes any effects of hypoxia on the bacterial expression of invasin, however, we cannot discount the possibility that such an effect may exist and contribute to the decrease in internalization. Furthermore, two other loci have been identified in *Y*. *enterocolitica*, Ail and YadA that contribute to cell surface attachment to host cells [17,58–60]. *Y*. *pseudotuberculosis* strains lacking invasin were still able to associate with Caco-2 cells although internalization was abolished [50].

Our results show that bacterial internalization is decreased under hypoxia and abolished in the absence of bacterial invasin or active β_1_ integrins. Furthermore, reduced levels of phosphorylated FAK (p-FAK) were confirmed in hypoxia pre-incubated cells infected with *Y*. *enterocolitica*. Since invasin-mediated binding of β_1_ integrins promotes recruitment of tyrosine kinases like FAK [39], these data support our hypothesis that the invasin-integrin-mediated internalization of *Y*. *enterocolitica* is altered under hypoxia.

The GI tract has been described to be in a state of constant, low grade inflammation associated with hypoxia, with intestinal epithelial cells playing a pivotal role in mucosal immunity and response to this inflammation [61]. Furthermore, chronic inflammation can be found in cases of inflammatory bowel disease (IBD), which has also been shown to result in hypoxic conditions [62]. In fact, intestinal epithelial cells have revealed a strong resilience to low oxygen conditions and have efficiently adapted to this physiological state [63]. Among these adaptation mechanisms is the accumulation of HIF-1, a transcription factor consisting of two subunits: the oxygen regulated α- and a constitutively expressed β-subunit [42,64]. HIF-1α has now been shown to be present ubiquitously in human tissues and plays an important role in the cellular adaptation to hypoxia [65]. Under normoxic conditions, the HIF-1α subunit is rapidly degraded by ubiquitination and subsequent proteosomal degradation mediated by oxygen- and iron- dependent prolyl hydroxylases (PHDs) [66,67]. The HIF-prolyl hydroxylases are dioxygenase enzymes that require oxygen and 2-oxoglutarate, rendering them key oxygen sensors [68,69]. Hypoxic conditions allow for HIF-1α accumulation due to the interruption of its degradation pathway [70,71]. Following HIF-1α stabilization, it dimerizes with the HIF-1β subunit and subsequently binds to specific hypoxia response elements (HREs) on target genes [71–73]. HIF-1α binding regulates the transcription of several target genes that encode, among others, angiogenic factors, proliferation and survival factors, glucose transporters, glycolytic enzymes, and antimicrobial factors [74,75].

Here, we have shown that the decreased internalization of *Y*. *enterocolitica* in hypoxic-treated Caco-2 cells goes along with increased protein levels of HIF-1α. Furthermore, the pan-hydroxylase inhibitor DMOG that is commonly used to stabilize HIF-1α [43] shows a similar phenotype. These data lead to the hypothesis that HIF-1α may contribute to this process. However, an isoform of HIF-1α, HIF-2α, may also be involved in this mechanism since it shares several regulatory functions with HIF-1α and is subject to a similar oxygen-dependent degradation by prolyl hydroxylases [76]. Therefore, studies with genetically modified cells are needed to verify this hypothesis. Interestingly, several studies described an effect of hypoxia on integrin expression, and in fact, a binding site for the transcription factor HIF-1α has been found on the β_1_ integrin (ITGB1) gene promoter in colonic fibroblasts that results in a significant increase in ITGB1 induction under hypoxia [77,78]. Besides transcriptional regulation of ITGB1, however, many hypoxia-induced post-translational modifications have also been reported [78–80]. In renal epithelial cells, hypoxia results in over-activation of the calcium-dependent cysteine protease, calpain, which then leads to unrestrained cleavage of integrins [81,82]. In several cell types, hypoxia increased β_1_ integrin mRNA levels but hindered maturation and localization, thus resulting in decreased protein levels or activation [78,83,84]. Additionally, oxygen-dependent modifications of the host cytoskeleton significantly affect the paracellular permeability, intracellular transport and the general endocytic uptake of particles [7]. Finally, an exposure to hypoxia induces significant remodeling of the host cell membrane microdomains (lipid rafts) in alveolar epithelial cells [8]. Lipid rafts have been shown to function in cell signaling, intracellular membrane transport, cell adhesion and host-pathogen interactions [8,85,86]. Whether the hypoxia-induced changes in the host cytoskeleton and membrane microdomains play a role in decreased bacterial entry into epithelial cells remains unclear.

The intricate relationship between hypoxia, infection and inflammation has been thoroughly investigated and besides HIF-1α various transcription factors are involved in this cellular stress response pathways, including Nuclear Factor Kappa Beta (NFκB) and cAMP Responsive Element Binding protein (CREB), among many others [87,88]. Furthermore, many of these investigations have been performed *in vivo*, where all these factors contribute to the immune response during hypoxia and infection [1,3,89]. Future experiments with genetically modified cells will be directed to characterize the cellular pathways involved in the hypoxia-modulated internalization of *Y*. *enterocolitica* into Caco-2 cells.

Interestingly, a decrease of bacterial internalization under hypoxia was shown for *Shigella flexneri* into host epithelial cells in a predicted HIF-1α -dependent manner [90]. Moreover, *Pseudomonas aeruginosa* entry into alveolar cells was decreased under hypoxia and after DMOG treatment, confirming the role of HIF-1α in an *in vivo* pneumonia model [91]. However, there has been no mention of the involvement of the β_1_ integrins in the hypoxia-induced decrease in internalization. Furthermore, evaluation of HIF-1α stabilization by pharmacological inhibition of prolyl hydroxylases, namely the HIF-1-specific PHD inhibitor AKB-4924, have revealed an important role for HIF-1α in boosting the innate immune response of keratinocytes against skin infections [92] and of the intestinal epithelium in murine colitis [93]. In light of these results, understanding the effects of hypoxia on epithelial cells during infections offers a new potential for pharmacological interference and the use of HIF-1α as a therapeutic target [5,6,94].
